# Microbial biosynthesis of lactate esters

**DOI:** 10.1186/s13068-019-1563-z

**Published:** 2019-09-20

**Authors:** Jong-Won Lee, Cong T. Trinh

**Affiliations:** 1 Bredesen Center for Interdisciplinary Research and Graduate Education, University of Tennessee, Knoxville, TN USA; 2 Center for Bioenergy Innovation, Oak Ridge National Laboratory, Oak Ridge, TN USA; 3 Department of Chemical and Biomolecular Engineering, University of Tennessee, 1512 Middle Dr., DO#432, Knoxville, TN 37996 USA

**Keywords:** Ester, Lactate ester, Ethyl lactate, Isobutyl lactate, Acetate ester, Alcohol acyltransferase, Green solvent, Modular cell, *Escherichia coli*

## Abstract

**Background:**

Green organic solvents such as lactate esters have broad industrial applications and favorable environmental profiles. Thus, manufacturing and use of these biodegradable solvents from renewable feedstocks help benefit the environment. However, to date, the direct microbial biosynthesis of lactate esters from fermentable sugars has not yet been demonstrated.

**Results:**

In this study, we present a microbial conversion platform for direct biosynthesis of lactate esters from fermentable sugars. First, we designed a pyruvate-to-lactate ester module, consisting of a lactate dehydrogenase (*ldhA*) to convert pyruvate to lactate, a propionate CoA-transferase (*pct*) to convert lactate to lactyl-CoA, and an alcohol acyltransferase (*AAT*) to condense lactyl-CoA and alcohol(s) to make lactate ester(s). By generating a library of five pyruvate-to-lactate ester modules with divergent AATs, we screened for the best module(s) capable of producing a wide range of linear, branched, and aromatic lactate esters with an external alcohol supply. By co-introducing a pyruvate-to-lactate ester module and an alcohol (i.e., ethanol, isobutanol) module into a modular *Escherichia coli* (chassis) cell, we demonstrated for the first time the microbial biosynthesis of ethyl and isobutyl lactate esters directly from glucose. In an attempt to enhance ethyl lactate production as a proof-of-study, we re-modularized the pathway into (1) the upstream module to generate the ethanol and lactate precursors and (2) the downstream module to generate lactyl-CoA and condense it with ethanol to produce the target ethyl lactate. By manipulating the metabolic fluxes of the upstream and downstream modules through plasmid copy numbers, promoters, ribosome binding sites, and environmental perturbation, we were able to probe and alleviate the metabolic bottlenecks by improving ethyl lactate production by 4.96-fold. We found that AAT is the most rate-limiting step in biosynthesis of lactate esters likely due to its low activity and specificity toward the non-natural substrate lactyl-CoA and alcohols.

**Conclusions:**

We have successfully established the biosynthesis pathway of lactate esters from fermentable sugars and demonstrated for the first time the direct fermentative production of lactate esters from glucose using an *E. coli* modular cell. This study defines a cornerstone for the microbial production of lactate esters as green solvents from renewable resources with novel industrial applications.

## Background

Solvents are widely used as primary components of cleaning agents, adhesives, and coatings and in assisting mass and heat transfer, separation and purification of chemical processes [1]. However, these solvents are volatile organic compounds (VOCs) that contribute to ozone depletion and photochemical smog via free radical air oxidation and hence cause many public health problems such as eye irritation, headache, allergic skin reaction, and cancer [1, 2]. Thus, recent interest in the use of alternative green solvents is increasing to satisfy environmental regulation and compelling demand for the eco-friendly solvents derived from renewable sources [3, 4].

Lactate esters are platform chemicals that have a broad range of industrial applications in flavor, fragrance, and pharmaceutical industries [5]. These esters are generally considered as green solvents because of their favorable toxicological and environmental profiles. For instance, ethyl lactate is 100% biodegradable, non-carcinogenic, non-corrosive, low volatile, and unhazardous to human health and the environment [6]. Due to the unique beneficial properties of ethyl lactate, it has been approved as a Significant New Alternatives Policy (SNAP) solvent by the U.S. Environmental Protection Agency (EPA) and as food additives by the U.S. Food and Drug Administration (FDA) [6]. Recent technical and economic analysis conducted by the National Renewable Energy Laboratory (NREL) considers ethyl lactate to be one of the top 12 bioproducts [7].

In industrial chemical processes, lactate esters are currently produced by esterification of lactic acid with alcohols using homogenous catalysts (e.g., sulfuric acid, hydrogen chloride, and/or phosphoric acid) under high temperature reaction conditions [8]. However, use of strong acids as catalysts causes corrosive problems and often requires more costly equipment for process operation and safety. Furthermore, the esterification reactions are thermodynamically unfavorable (Δ*G *= + 5 kcal/mol) in aqueous solutions and often encounter significant challenge due to self-polymerization of lactate [9]. Alternatively, microbial catalysts can be harnessed to produce these esters from renewable and sustainable feedstocks in a thermodynamically favorable reaction (Δ*G* = − 7.5 kcal/mol) in an aqueous phase environment at room temperature and atmospheric pressure [10–16]. This reaction uses an alcohol acyltransferase (AAT) to generate an ester by condensing an alcohol and an acyl-CoA. AAT can catalyze a broad substrate range including (i) linear or branched short-to-long chain fatty alcohols [10, 11, 17], (ii) aromatic alcohols [18], and (iii) terpenols [19–22] as well as various fatty acyl-CoAs [11, 13]. To date, while microbial biosynthesis of the precursor metabolites for lactate esters have been well established such as lactate [13, 16, 23–27], lactyl-CoA [28–30], ethanol [31, 32], propanol [33], isopropanol [34], butanol [35], isobutanol [36], amyl alcohol [37], isoamyl alcohol [38], benzyl alcohol [39], 2-phenylethanol [40, 41], and terpenols [19–22], the direct microbial biosynthesis of lactate esters from fermentable sugars has not yet been demonstrated.

In this work, we aimed to demonstrate the feasibility of microbial production of lactate esters as green organic solvents from renewable resources. To enable the direct microbial biosynthesis of lactate esters from fermentable sugars, we first screened for an efficient AAT suitable for lactate ester production using a library of five pyruvate-to-lactate ester modules with divergent AATs. We next demonstrated direct fermentative biosynthesis of ethyl and isobutyl lactate esters from glucose by co-introducing a pyruvate-to-lactate ester module and an alcohol module (i.e., ethanol and isobutanol) into an engineered *Escherichia coli* modular cell. As a proof-of-study to improve ethyl lactate production, we employed a combination of metabolic engineering and synthetic biology approaches to dissect the pathway to probe and alleviate the potential metabolic bottlenecks.

## Results and discussion

### In vivo screening of efficient AATs critical for lactate ester biosynthesis

**Fig. 1:**
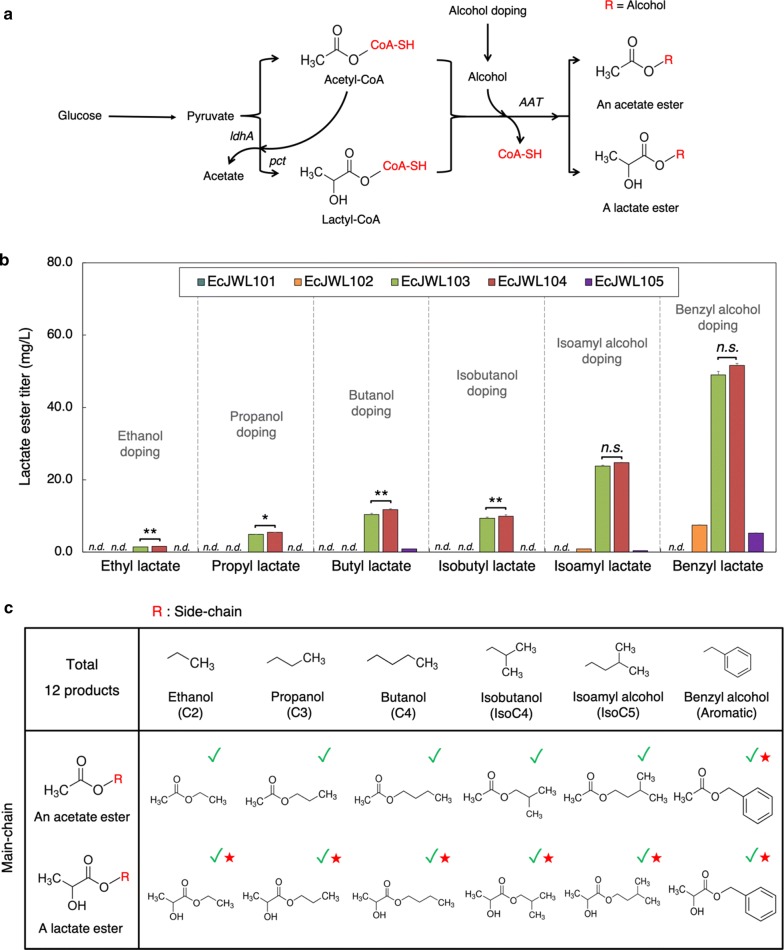
In vivo characterization of various alcohol acyltransferases for biosynthesis of lactate esters. **a** Biosynthesis pathways of lactate and acetate esters with external supply of alcohols. **b** Ester production of EcJW101, EcJW102, EcJW103, EcJW104, and EcJW105 harboring *ATF1*, *ATF2*, *SAAT*, *VAAT*, and *atfA*, respectively in high cell density cultures with various alcohol doping. Each error bar represents 1 standard deviation (s.d., *n *= *3*). Symbols: *n.d.* not detected, *n.s.* not significant, *p < 0.073, and **p < 0.013 (Student’s t-test). **c** The library of esters produced. Green check marks indicate the esters produced in this study while red star marks indicate the esters produced for first time in engineered strains

The substrate specificity of AATs is critical to produce target esters [13]. For example, ATF1 exhibits substrate preference for biosynthesis of acyl (C4–C6) acetates while SAAT and VAAT prefer biosynthesis of ethyl (C2–C6) acylates. Even though both SAAT and VAAT are derived from the same strawberry genus, they also show very distinct substrate preferences; specifically, SAAT prefers longer (C4–C6) acyl-CoAs whereas VAAT prefers shorter (C2–C4) acyl-CoAs. To date, none of AATs have been tested for lactate ester biosynthesis. Thus, to enable lactate ester biosynthesis, we began with identification of the best AAT candidate. We designed, constructed, and characterized a library of five pyruvate-to-lactate ester modules (pJW002-006) carrying five divergent AATs including ATF1, ATF2, SAAT, VAAT, and AtfA, respectively. AtfA was used as a negative control because it prefers long-chain acyl-CoAs (C14–C18) and alcohols (C14–C18) [42]. For characterization, 2 g/L of ethanol, propanol, butanol, isobutanol, isoamyl alcohol, and benzyl alcohol were added to culture media with 0.5 mM of IPTG for pathway induction to evaluate biosynthesis of six different lactate esters including ethyl lactate, propyl lactate, butyl lactate, isobutyl lactate, isoamyl lactate, and benzyl lactate, respectively, in high cell density cultures (Fig. 1a).

The results show that most of the strains could produce different types of lactate esters with external supply of alcohols (Fig. 1b, c). EcJW104 achieved the highest titer of lactate esters in all cases, producing 1.59 ± 0.04 mg/L of ethyl lactate, 5.46 ± 0.25 mg/L of propyl lactate, 11.75 ± 0.43 mg/L of butyl lactate, 9.92 ± 0.08 mg/L of isobutyl lactate, 24.73 ± 0.58 mg/L of isoamyl lactate, and 51.59 ± 2.09 mg/L of benzyl lactate in ethanol, propanol, butanol, isobutanol, isoamyl alcohol, and benzyl alcohol doping, respectively. The lactate ester biosynthesis of EcJW104 exhibited different alcohol substrate preference in the following order: benzyl alcohol > isoamyl alcohol > butanol > isobutanol > propanol > ethanol (Fig. 1b, Additional file 1: Table S2).

Due to the presence of endogenous acetyl-CoA, we also produced acetate esters in addition to lactate esters (Fig. 1). Among the strains, EcJW101 achieved the highest titers of acetate esters in all cases, producing 115.52 ± 4.83 mg/L of ethyl acetate, 801.62 ± 33.51 mg/L of propyl acetate, 1017.90 ± 20.21 mg/L of butyl acetate, 1210.40 ± 24.83 mg/L of isobutyl acetate, 692.73 ± 7.65 mg/L of isoamyl acetate, and 1177.98 ± 45.72 mg/L of benzyl acetate in ethanol, propanol, butanol, isobutanol, isoamyl alcohol, and benzyl alcohol doping, respectively. EcJW101 showed different alcohol substrate preference for the acetate ester biosynthesis in the following order: isobutanol > benzyl alcohol > butanol > propanol > isoamyl alcohol > ethanol (Additional file 1: Table S2).

Taken altogether, VAAT and ATF1 are the most suitable AATs for biosynthesis of lactate esters and acetate esters, respectively. Among the library of 12 esters (Fig. 1c), seven of these esters, including ethyl lactate, propyl lactate, butyl lactate, isobutyl lactate, isoamyl lactate, benzyl lactate, and benzyl acetate, were demonstrated for in vivo production in microbes for the first time. EcJW104 that harbors the pyruvate-to-lactate module with *VAAT* could produce 6 out of 6 target lactate esters including ethyl, propyl, butyl, isobutyl, isoamyl, and benzyl lactate. Since EcJW104 achieved the highest titer of lactate esters in all cases, it was selected for establishing the biosynthesis pathway of lactate esters from glucose.

### Establishing the lactate ester biosynthesis pathways

We next demonstrated direct fermentative production of lactate esters from glucose using the best VAAT candidate. We focused on the biosynthesis of ethyl and isobutyl lactate esters. We designed the biosynthesis pathways for ethyl and isobutyl lactate by combining the pyruvate-to-lactate ester module (pJW005) with the ethanol (pCT24) and isobutanol (pCT13) modules, respectively. By co-transforming pJW005/pCT24 and pJW005/pCT13 into the modular cell EcDL002, we generated the production strains, EcJW201 and EcJW202, for evaluating direct conversion of glucose to ethyl and isobutyl lactate esters, respectively.

**Fig. 2:**
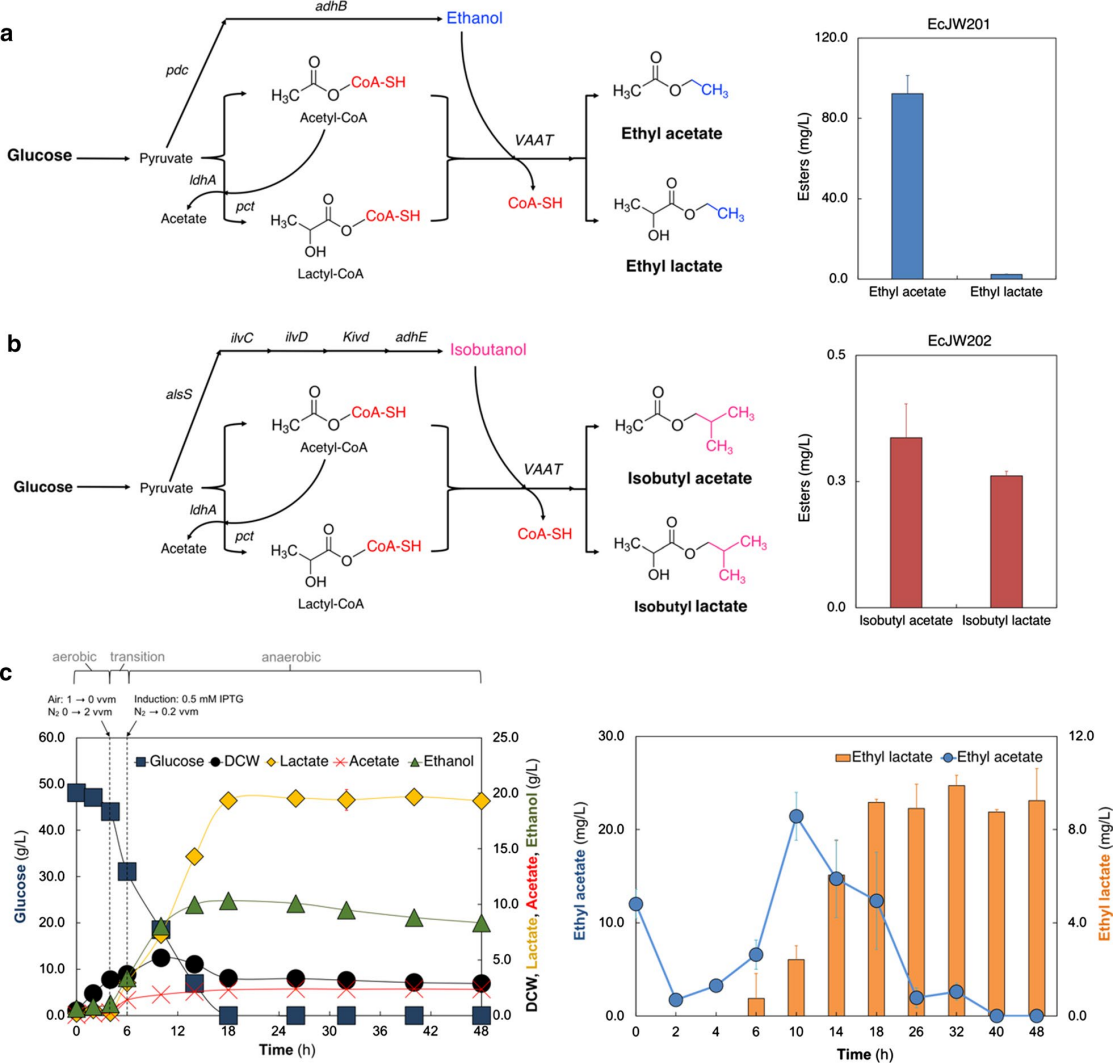
Design, construction, and validation of the lactate ester biosynthesis pathways in *E. coli*. **a** Engineered biosynthesis pathway of ethyl lactate from glucose and its production in high cell density culture of EcJW201. **b** Engineered biosynthesis pathway of isobutyl lactate from glucose and its production in high cell density culture of EcJW202. In **a** and **b**, all of the strains were induced at 0 h with 0.5 mM IPTG. Each error bar represents 1 s.d. (*n* = 3). **c** Production of ethyl lactate from glucose in pH-controlled batch fermentation of EcJW201. The strain was induced at 6 h with 0.5 mM IPTG. Each error bar represents 1 s.d. (*n *= 2)

We characterized EcJW201 and EcJW202 together with the parent strain, EcDL002, as a negative control in high cell density cultures. The results show EcJW201 and EcJW202 produced ethyl (Fig. 2a) and isobutyl (Fig. 2b) lactate from glucose, respectively, while the negative control strain EcDL002 could not. Consistently, the expressions of metabolic enzymes of the ethyl and isobutyl lactate pathways were confirmed in EcJW201 and EcJW202, respectively, by SDS-PAGE analysis (Additional file 2: Figure S1). During 24 h fermentation, EcJW201 produced 2.24 ± 0.28 mg/L of ethyl lactate with a specific productivity of 0.04 ± 0.00 mg/gDCW/h while EcJW202 produced 0.26 ± 0.01 mg/L of isobutyl lactate with a specific productivity of 0.01 ± 0.00 mg/gDCW/h. In addition to ethyl or isobutyl lactate biosynthesis, EcJW201 also produced 92.25 ± 9.20 mg/L of ethyl acetate while EcJW202 generated 1.36 ± 0.74 mg/L of ethyl acetate and 0.34 ± 0.07 mg/L of isobutyl acetate (Additional file 1: Table S3A). Taken altogether, the direct microbial synthesis of lactate esters from fermentable sugar was successfully demonstrated. Since the lactate ester production was low, the next logical step was to identify and alleviate the key pathway bottlenecks for enhanced lactate ester biosynthesis. As proof-of-principle, we focused on optimization of the ethyl lactate production as presented in the subsequent sections.

### Identifying and alleviating key bottlenecks of the ethyl lactate biosynthesis pathway

#### Evaluating the biosynthesis of ethyl lactate in pH-controlled fermentation as a basis to identify potential pathway bottlenecks

In an attempt to identify the key bottlenecks of the ethyl lactate biosynthesis pathway, we characterized EcJW201 in pH-controlled bioreactors. The results show that EcJW201 produced 9.17 ± 0.12 mg/L of ethyl lactate with a specific productivity of 0.15 ± 0.02 mg/gDCW/h and a yield of 0.19 ± 0.00 mg/g glucose (Fig. 2c, Additional file 1: Table S3B) in 18 h. Under pH-controlled fermentation, EcJW201 achieved 4.09-fold (from 2.24 ± 0.28 to 9.17 ± 0.12 mg/L), 3.75-fold (from 0.04 ± 0.00 to 0.15 ± 0.02 mg/gDCW/h), and 19-fold (from 0.01 ± 0.00 to 0.19 ± 0.00 mg/g glucose) improvement in titer, specific productivity, and yield, respectively, as compared to the strain performance in the high cell density culture. It is interesting to observe that ethyl acetate was first produced then consumed after 10 h, which is likely due to the endogenous esterase of *E. coli* as observed in a recent study [15]. Different from ethyl acetate, we did not observe ethyl lactate degradation during fermentation, especially after glucose was depleted. Even though the strain performance in pH-controlled bioreactors was enhanced by increased supply of precursor metabolites (19.35 ± 0.29 g/L of lactate and 10.31 ± 0.41 g/L of ethanol, Additional file 1: Table S3B) from higher concentration of carbon source, the titer of ethyl lactate did not increase during the fermentation. This result suggests that (i) rate-limiting conversion of lactate into lactyl-CoA by Pct and/or condensation of lactyl-CoA with an ethanol by VAAT and/or (ii) toxicity of ethyl lactate on *E. coli* health might have limited lactate ester biosynthesis. Therefore, to enhance ethyl lactate production, it is important to elucidate and alleviate these identified potential bottlenecks.

#### Ethyl lactate exhibited minimal toxicity on cell growth among lactate esters

To determine whether lactate esters inhibited cell growth and hence contributed to low lactate ester production, we cultured the parent strain, EcDL002, in a microplate reader with or without supply of various concentrations of lactate esters including ethyl, propyl, butyl, isobutyl, isoamyl, or benzyl lactate. The results show that ethyl lactate was the least toxic among the six lactate esters characterized where the growth rate (0.47 ± 0.04 L/h) and cell titer (OD_600_ = 0.42 ± 0.03) decreased by 6% and 10%, respectively, upon cell exposure to 5 g/L ethyl lactate. On the other hand, isoamyl lactate was the most toxic among the lactate esters, where cell exposure to only 0.5 g/L ester resulted in 18% and 15% reduction in the growth rate (0.41 ± 0.02 L/h) and OD_600_ (0.40 ± 0.03), respectively (Additional file 2: Figure S2A). The toxicity of lactate esters can be ranked in the following order: isoamyl lactate > benzyl lactate > butyl lactate > isobutyl lactate > propyl lactate > ethyl lactate. There existed a positive correlation between the logP values of lactate esters and their degrees of toxicity (Additional file 2: Figure S2B). This result was consistent with literature, illustrating that increasing toxicity of esters is highly correlated with increasing chain length of alcohol moieties that can severely disrupt cell membrane [43]. It should be note that since *E. coli* can effectively secrete short-chain esters [10], external exposure of cells to lactate esters in our experiment design is sufficient to probe the potential toxicity caused by endogenous production of these esters. Taken altogether, ethyl lactate is the least toxic and was not likely the main reason for the low production of ethyl lactate observed. It was likely the downstream pathway, responsible for conversion of lactate into lactyl-CoA by Pct and/or condensation of lactyl-CoA with ethanol by VAAT, might have been contributed to the inefficient ethyl lactate biosynthesis.

##### Downstream pathway of the lactate ester biosynthesis is the key bottleneck

**Fig. 3:**
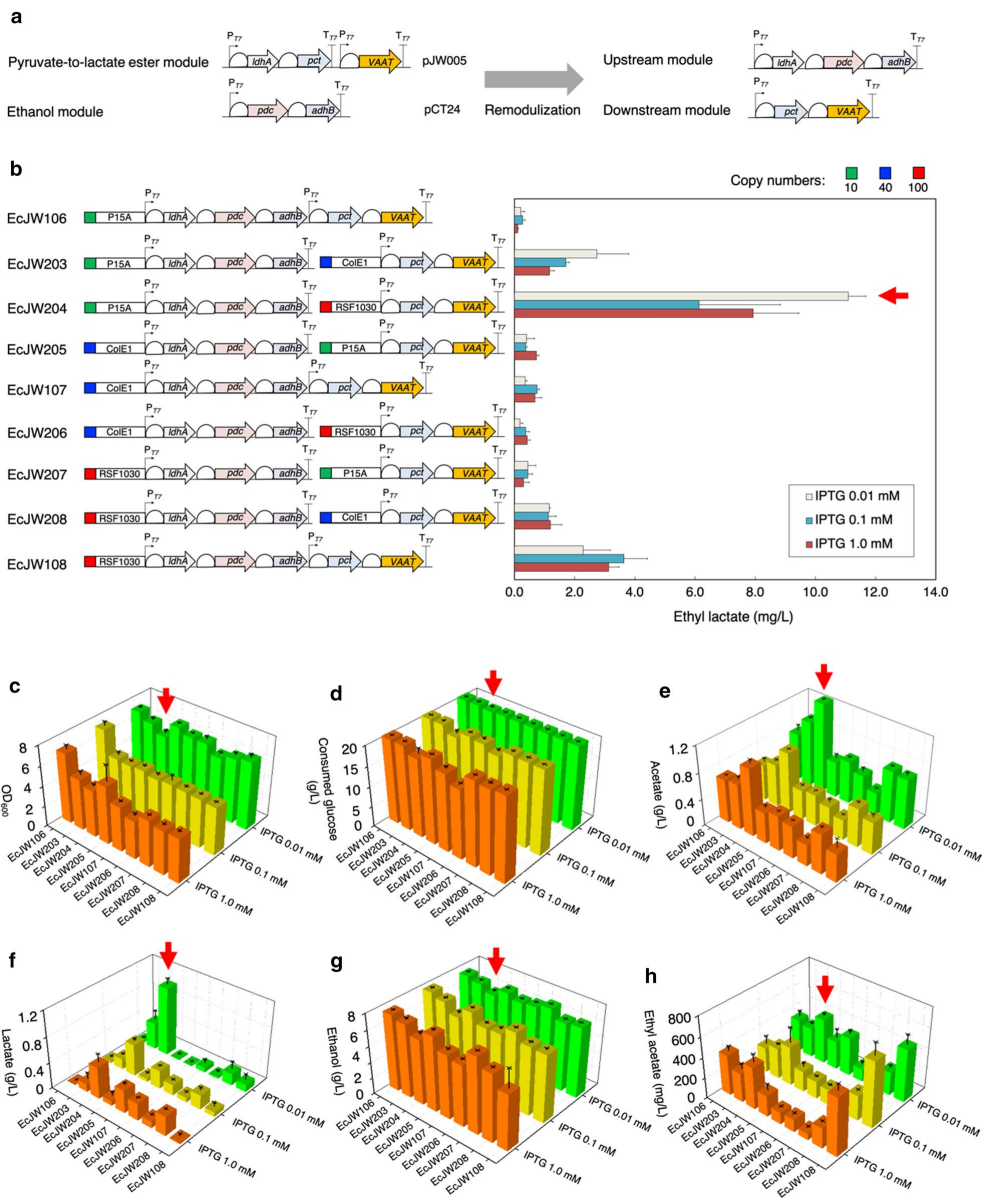
Combinatorial modular pathway optimization for enhanced ethyl lactate biosynthesis by varying plasmid copy number. **a** Re-modularization of the ethyl lactate biosynthesis pathway. Pyruvate-to-lactate ester and ethanol modules were re-modulated into upstream and downstream modules using plasmids with different copy numbers. **b** Ethyl lactate production, **c** OD_600_, **d** Consumed glucose, **e** Acetate, **f** Lactate, **g** Ethanol, and **h** Ethyl acetate of EcJW106-108 and EcJW203-208 in high cell density cultures induced with various concentrations of IPTG. Green rectangle: low copy number plasmid (10); P15A: origin of pACYCDuet-1; blue rectangle: medium copy number plasmid (40); ColE1: origin of pETDuet-1; red rectangle: high copy number plasmid (100); RSF1030: origin of pRSFDuet-1; P_T7_: T7 promoter; T_T7_: T7 terminator. All of the strains were induced at 0 h with 0.01, 0.1, or 1.0 mM IPTG, respectively. Each error bar represents 1 s.d. (*n *=* 3*). Red arrows indicate the selected strain with an optimum concentration of IPTG for the further studies

To identify and alleviate the ethyl lactate biosynthesis pathway, we re-modularized it with two new parts: (i) the upstream module carrying *ldhA*, *pdc*, and *adhB* for production of lactate and ethanol from sugar and (ii) the downstream module carrying *pct* and *VAAT* for converting lactate into lactyl-CoA and condensing lactyl-CoA and ethanol (Fig. 3a). We controlled metabolic fluxes of these modules by manipulating their plasmid copy numbers and levels of promoter induction with IPTG. By introducing the plasmids pJW007-015 into EcDL002, we generated the strains EcJW106-108 and EcJW203-208, respectively (Fig. 3b). To evaluate the performance of these constructed strains for ethyl lactate production, we characterized them in high cell density cultures induced with various concentrations of IPTG (0.01, 0.1, and 1.0 mM).

The results show that EcJW204, carrying the upstream module with a low copy number plasmid (P15A origin) and the downstream module with a high copy number plasmid (RSF1030 origin) induced by 0.01 mM of IPTG, achieved the highest titer of ethyl lactate. As compared to EcJW201, EcJW204 achieved 4.96-fold (an increase from 2.24 to 11.10 ± 0.58 mg/L), 5.50-fold (from 0.04 ± 0.00 to 0.22 ± 0.02 mg/gDCW/h), and 54.0-fold (from 0.01 ± 0.00 to 0.54 ± 0.04 mg/g glucose) improvement in titer, specific productivity, and yield of ethyl lactate, respectively (Fig. 3b, Additional file 1: Table S5). Upon IPTG induction at 24 h, we observed the reduced cell growth of the host strains with use of high concentration of IPTG (Fig. 3c, Additional file 1: Table S4), suggesting that they suffered from metabolic burden due to overexpression of multiple enzymes [44] and also explaining why use of low concentration of IPTG can help yield better production of ethyl lactate.

Although EcJW204 showed better performance in ethyl lactate production than EcJW201, the accumulation of lactate and ethanol was still observed (Fig. 3f, g, Additional file 1: Table S4), indicating the pathway bottleneck remained. In particular, the downstream module flux was outcompeted by the upstream module flux and hence failed to turn over the precursor metabolites quickly enough. This result helps explain why a combination of the upstream module (for producing lactate and ethanol from sugar) with a low copy number plasmid and the downstream module (for converting lactate into lactyl-CoA and condensing lactyl-CoA and ethanol) with a high copy number plasmid outperformed eight other combinations. Notably, the best ethyl lactate producer EcJW204 achieved the highest lactate and lowest ethanol production among the nine characterized strains (Fig. 3f, g, Additional file 1: Table S4), suggesting redistribution of the carbon flux from ethanol to lactate likely helped improve ethyl lactate production. Thus, we hypothesized that redistribution of the carbon source from ethanol to lactate would help to improve ethyl lactate production. To test this hypothesis, we first examined whether (i) downregulation of the ethanol flux of the upstream module enabled redistribution of the carbon flow from ethanol to lactate and (ii) this redistribution could improve ethyl lactate production before proceeding to investigate the potential bottleneck of downstream module.

##### High ethanol synthesis of the upstream module was critical for ethyl lactate biosynthesis due to low specificity and activity of AAT

**Fig. 4:**
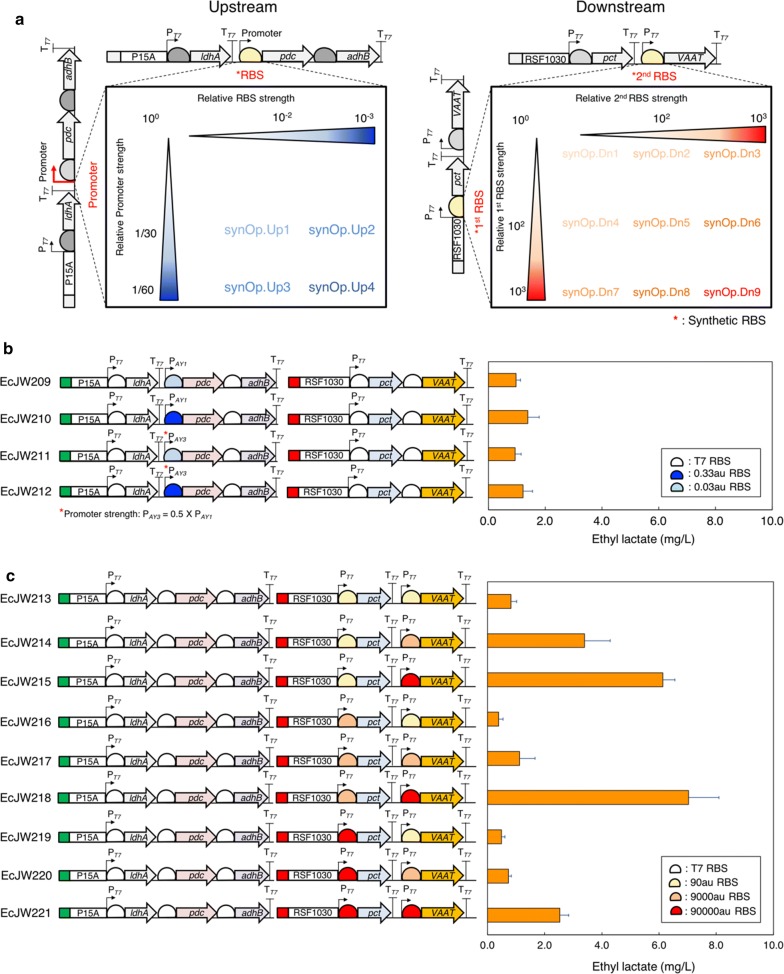
Probing and alleviating the potential metabolic bottlenecks of the upstream or downstream modules of EcJW204 by varying the strength of promoters and/or ribosome binding sites. **a** Design of synthetic operons for the upstream and downstream modules. For the upstream module, the T7 promoter in MCS2 and the RBS between T7 promoter in MCS2 and the start codon of *pdc* were replaced with the combination of P_AY1_ or P_AY3_ promoter and 0.3 or 0.03 a.u. RBS. For the downstream module, the RBS between T7 promoter in MCS1 and the start codon of *pct* gene and the RBS between T7 promoter in MCS2 and the start codon of *VAAT* gene were replaced with the combination of 90, 9000, or 90000 a.u. RBS and 90, 9000, or 90000au RBS, respectively. Production of ethyl lactate in high cell density cultures of **b** EcJW209-212 and **c** EcJW213-221. Green rectangle: low copy number plasmid (10); P15A: origin of pACYCDuet-1; red rectangle: high copy number plasmid (100); RSF1030: origin of pRSFDuet-1; P_T7_: T7 promoter; T_T7_: T7 terminator. All of the strains were induced at 0 h with 0.01 mM IPTG. Each error bar represents 1 s.d. (*n *= 3)

To downregulate the ethanol flux of the upstream module, we first reconfigured pJW007, the upstream module of the best performer EcJW204, with a library of two weaker promoters and four weaker synthetic RBSs (Fig. 4a, Additional file 2: Figure S3A), resulting in four new upstream modules (pJW019-022). By introducing each newly constructed upstream module into EcDL002 together with the downstream module pJW012 used in EcJW204, we next generated the strains EcJW209-212 and characterized them in high cell density cultures induced with 0.01 mM IPTG.

The results show that while the carbon flux was successfully redistributed from ethanol to lactate, resulting in 5.97- to 6.92-fold decrease in ethanol production (from 8.30 ± 0.17 to 1.39 ± 0.10 ~ 1.20 ± 0.01 g/L) and 1.67- to 2.59-fold increase in lactate production (from 1.06 ± 0.09 to 1.77 ± 0.37 g/L ~ 2.75 ± 0.09 g/L) (Additional file 1: Table S6A), the ethyl lactate production was reduced by 7.99- to 11.81-fold in ethyl lactate production (from 11.10 ± 0.58 to 1.39 ± 0.40 ~ 0.94 ± 0.22 mg/L) in all four characterized strains as compared to that of EcJW204 (Fig. 4b, Additional file 1: Table S6B). This result suggests that a high level of ethanol is critical for VAAT to produce ethyl lactate.

**Fig. 5:**
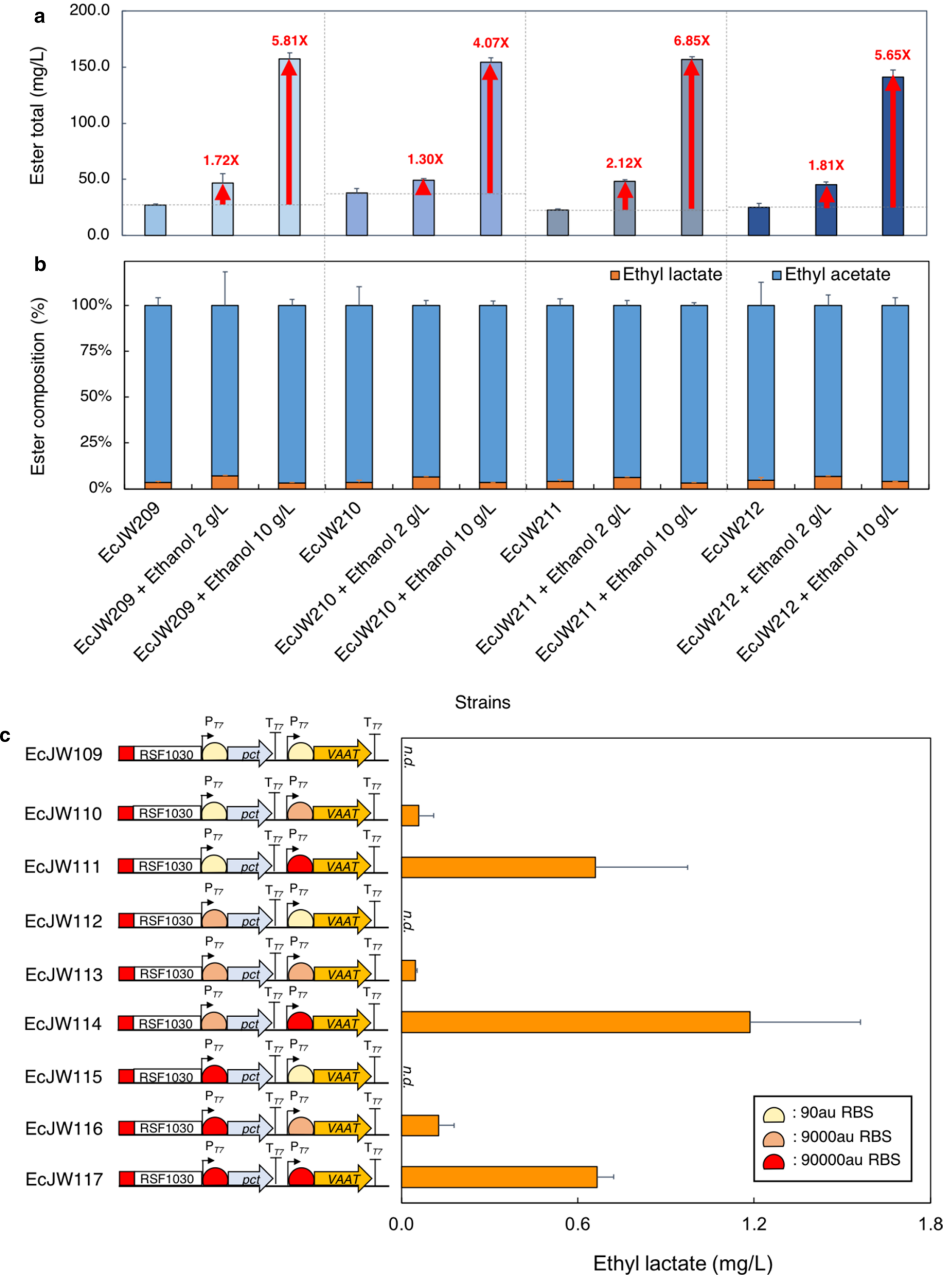
**a** Total esters and **b** Composition of total esters produced in high cell density cultures of EcJW209-212 with or without addition of ethanol. **c** Ethyl lactate production of EcJW109-117 with addition of 2 g/L of lactate and ethanol. Red rectangle: high copy number plasmid (100); RSF1030: origin of pRSFDuet-1; P_T7_: T7 promoter; T_T7_: T7 terminator. All of the strains were induced at 0 h with 0.01 mM IPTG. Each error bar represents 1 s.d. (*n *= 3)

To support this conclusion, we evaluated the effect of external ethanol supply on production of ethyl esters in high cell density cultures of EcJW209-212 induced with 0.01 mM IPTG. Indeed, with external ethanol supply, we observed enhanced production of both ethyl lactate and ethyl acetate in EcJW209-212. In specific, with addition of 2 g/L of ethanol, the ethyl lactate and ethyl acetate production increased by 2.27- to 3.33-fold (from 1.39 ± 0.40 to 3.15 ± 0.15 mg/L ~ from 0.98 ± 0.15 to 3.26 ± 0.26 mg/L) and 1.27- to 2.07-fold (from 36.46 ± 3.86 to 46.22 ± 1.33 mg/L ~ from 21.96 ± 0.84 to 45.40 ± 1.20 mg/L), respectively (Additional file 1: Table S6). Further addition of ethanol up to 10 g/L improved the ethyl lactate and ethyl acetate production by 3.78- to 5.26-fold (from 1.39 ± 0.40 to 5.26 ± 0.27 mg/L ~ from 0.94 ± 0.15 mg/L to 4.49 ± 0.41 mg/L) and 4.09- to 6.92-fold (from 36.46 ± 3.86 to 148.97 ± 3.80 mg/L ~ from 21.96 ± 0.84 mg/L to 151.87 ± 2.34 mg/L), respectively (Additional file 1: Table S6). Interestingly, while the total titer of ethyl esters increased with the increasing addition of ethanol (Fig. 5a), the proportion of ethyl lactate in the total ester slightly increased in the range of 3.2–7.0% (Fig. 5b), suggesting that VAAT prefers acetyl-CoA over lactyl-CoA with ethanol as a co-substrate. Notably, we observed a strong linear correlation between ethyl esters production and the amount of added ethanol (i.e., for ethyl lactate, *R*^2^ = 0.85–0.94; for ethyl acetate, *R*^2^ = 0.99–1.00) (Additional file 2: Figure S4A). The results revealed that abundant availability of ethanol is essential to achieve high production of ethyl esters, indicating the main reason for the improved ethyl lactate production in EcJW204 was most likely due to the upregulation of downstream module with a high copy number plasmid.

##### AAT was the most rate-limiting step of the downstream module

To determine whether Pct for conversion of lactate to lactyl-CoA or VAAT for condensation of lactyl-CoA and an alcohol was the most rate-limiting step of the downstream module, we redesigned and constructed nine downstream modules (pJW027-035) derived from pJW012 of the best performer EcJW204 using a combination of three synthetic RBSs for Pct expression (synRBS_pct#1-3_) and three synthetic RBSs for VAAT expression (synRBS_VAAT#1-3_) (Fig. 4a, Additional file 2: Figure S3B). We introduced each newly constructed downstream module into EcDL002 together with the original upstream module (pJW007) used in EcJW204 to generate EcJW213-221. Then, we characterized the constructed strains in high cell density cultures induced with 0.01 mM IPTG.

The results show that the strains harboring the stronger RBSs for VAAT expression achieved the higher titers of ethyl lactate and ethyl acetate regardless of the RBS strengths for Pct expression (Fig. 4c, Additional file 1: Table S7). There is a strong linear correlation between ethyl ester production and the strength of RBS for VAAT expression (Additional file 2: Figure S4B). To further validate these results without the influence of the upstream module, we additionally constructed the strains EcJW109-117 by introducing nine individual downstream modules (pJW027-035) into EcDL002 and then characterized these strains in high cell density cultures with addition of 2 g/L of lactate, 2 g/L of ethanol, and 0.01 mM of IPTG. We could observe the same strong linear correlation between ethyl ester production and high VAAT expression without the upstream module (Fig. 5c).

Taken altogether, these results suggest that VAAT not Pct was the most rate-limiting step of the downstream module of the ethyl lactate biosynthesis pathway. In specific, a combination of low affinity toward lactyl-CoA and ethanol of VAAT contributed to low ethyl lactate biosynthesis. Further studies on discovery of novel AATs, exhibiting high activity toward lactyl-CoA and alcohols but not acetyl-CoA, together with rational protein engineering of these enzymes would be warranted for improving lactate ester production.

In principle, the lactate ester platform can be controlled to produce enantiomers with broad industrial applications. Since the endogenous *E. coli* d-lactate dehydrogenase (LdhA) was overexpressed in the *ldhA*-deficient modular cell of our study, it is anticipated that d-(−)-lactate and the associated d-(−)-lactate esters were mainly produced. To date, production of optically pure d-(−)- [23] and l-(+)-form [26] of lactate from glucose in *E. coli* [25] has been well established. In addition, *pct* from *C. propionicum* [28] and *Megasphaera elsdenii* [29, 30] has been used for converting d-(−)-lactate into d-(−)-lactyl-CoA in polylactic acid (PLA) production in *E. coli* and their catalytic activity toward l-(+)-lactate has also been demonstrated [45, 46]. Thus, by combining stereospecific Ldh and Pct enzymes together with AATs, it is highly feasible to extend our lactate ester platform for microbial production of stereospecific lactate esters from renewable resources.

## Conclusions

In this study, we have successfully developed a microbial lactate ester production platform and demonstrated for the first time the microbial biosynthesis of lactate esters directly from fermentable sugars in an *E. coli* modular cell. This study defines a cornerstone for the microbial production of lactate esters as green solvents from renewable resources with novel industrial applications.

## Methods

### Strain construction

The list of strains used in this study is presented in Table 1. For molecular cloning, *E. coli* TOP10 strain was used. To generate the lactate ester production strains, the modules, including (i) the pyruvate-to-lactate ester modules (pJW002-006), (ii) the upstream and/or downstream modules (pJW007-pJW028), and (iii) the alcohol modules (pCT24 or pCT13), were transformed into the engineered modular *E. coli* chassis cell, EcDL002 [10] via electroporation [47].

**Table 1:** A list of strains used in this study

Strains	Genotypes	Sources
*E. coli* TOP10	F-*mcrA* Δ(*mrr*-*hsd*RMS-*mcr*BC) Φ80*lac*ZΔM15 Δ*lac*X74 *rec*A1 *ara*D139 Δ(*ara leu*) 7697 *gal*U *gal*K *rps*L (StrR) *end*A1 *nup*G	Invitrogen
*E. coli* MG1655	F^−^ λ^−^	ATCC 47076
*Clostridium propionicum*	Wildtype	ATCC 25522
EcDL002	TCS083 (λDE3) Δ*fadE*	[10]
EcJW101	EcDL002/pJW002; amp^R^	This study
EcJW102	EcDL002/pJW003; amp^R^	This study
EcJW103	EcDL002/pJW004; amp^R^	This study
EcJW104	EcDL002/pJW005; amp^R^	This study
EcJW105	EcDL002/pJW006; amp^R^	This study
EcJW201	EcDL002/pJW005 pCT24; amp^R^ kan^R^	This study
EcJW202	EcDL002/pJW005 pCT13; amp^R^ kan^R^	This study
EcJW106	EcDL002/pJW013; cm^R^	This study
EcJW203	EcDL002/pJW007 pJW011; cm^R^ amp^R^	This study
EcJW204	EcDL002/pJW007 pJW012; cm^R^ kan^R^	This study
EcJW205	EcDL002/pJW008 pJW010; cm^R^ amp^R^	This study
EcJW107	EcDL002/pJW014; amp^R^	This study
EcJW206	EcDL002/pJW008 pJW012; amp^R^ kan^R^	This study
EcJW207	EcDL002/pJW009 pJW010; cm^R^ kan^R^	This study
EcJW208	EcDL002/pJW009 pJW011; amp^R^ kan^R^	This study
EcJW108	EcDL002/pJW015; kan^R^	This study
EcJW209	EcDL002/pJW019 pJW012; cm^R^ kan^R^	This study
EcJW210	EcDL002/pJW020 pJW012; cm^R^ kan^R^	This study
EcJW211	EcDL002/pJW021 pJW012; cm^R^ kan^R^	This study
EcJW212	EcDL002/pJW022 pJW012; cm^R^ kan^R^	This study
EcJW213	EcDL002/pJW007 pJW027; cm^R^ kan^R^	This study
EcJW214	EcDL002/pJW007 pJW028; cm^R^ kan^R^	This study
EcJW215	EcDL002/pJW007 pJW029; cm^R^ kan^R^	This study
EcJW216	EcDL002/pJW007 pJW030; cm^R^ kan^R^	This study
EcJW217	EcDL002/pJW007 pJW031; cm^R^ kan^R^	This study
EcJW218	EcDL002/pJW007 pJW032; cm^R^ kan^R^	This study
EcJW219	EcDL002/pJW007 pJW033; cm^R^ kan^R^	This study
EcJW220	EcDL002/pJW007 pJW034; cm^R^ kan^R^	This study
EcJW221	EcDL002/pJW007 pJW035; cm^R^ kan^R^	This study
EcJW109	EcDL002/pJW027; kan^R^	This study
EcJW110	EcDL002/pJW028; kan^R^	This study
EcJW111	EcDL002/pJW029; kan^R^	This study
EcJW112	EcDL002/pJW030; kan^R^	This study
EcJW113	EcDL002/pJW031; kan^R^	This study
EcJW114	EcDL002/pJW032; kan^R^	This study
EcJW115	EcDL002/pJW033; kan^R^	This study
EcJW116	EcDL002/pJW034; kan^R^	This study
EcJW117	EcDL002/pJW035; kan^R^	This study

### Plasmid construction

The list of plasmids and primers used in this study are presented in Tables 2 and 3, respectively. Pathway construction includes pyruvate-to-lactate ester modules and a library of upstream and downstream modules with various plasmid copy numbers, promoters, and ribosome binding sites (RBSs).

**Table 2:** A list of plasmids used in this study

Plasmids	Genotypes	Sources
pACYCDuet-1	Two sets of MCS, T_7_ promoter, P15A ori; cm^R^	Novagen
pETDuet-1	Two sets of MCS, T_7_ promoter, ColE1 ori; amp^R^	Novagen
pRSFDuet-1	Two sets of MCS, T_7_ promoter, RSF1030 ori; kan^R^	Novagen
pETite*	T_7_ promoter, pBR322 ori; kan^R^	[10]
pCT24	pETite* P_T7_::*pdc*::*adhB*::T_T7_; kan^R^	[10]
pCT13	pCOLA P_T7_::*alsS*::*ilvC*::*ilvD*-P_T7_::*kivd*::*adhE*::T_T7_; kan^R^	[57]
pDL004	pETite* *ATF1*; kan^R^	[13]
pDL005	pETite* *ATF2*; kan^R^	[13]
pDL001	pETite* *SAAT*; kan^R^	[13]
pDL006	pETite* *VAAT*; kan^R^	[13]
pCT16	pETite* *atfA*; kan^R^	[58]
pJW001	pETite* P_T7_::*ldhA*::*pct*::T_T7_; amp^R^	This study
pJW002	pJW001 P_T7_::*ldhA*::*pct*-P_T7_::*ATF1*::T_T7_; amp^R^	This study
pJW003	pJW001 P_T7_::*ldhA*::*pct*-P_T7_::*ATF2*::T_T7_; amp^R^	This study
pJW004	pJW001 P_T7_::*ldhA*::*pct*-P_T7_::*SAAT*::T_T7_; amp^R^	This study
pJW005	pJW001 P_T7_::*ldhA*::*pct*-P_T7_::*VAAT*::T_T7_; amp^R^	This study
pJW006	pJW001 P_T7_::*ldhA*::*pct*-P_T7_::*atfA*::T_T7_; amp^R^	This study
pJW007	pACYCDuet-1 P_T7_::*ldhA*::*pdc*::*adhB*::T_T7_; cm^R^	This study
pJW008	pETDuet-1 P_T7_::*ldhA*::*pdc*::*adhB*::T_T7_; amp^R^	This study
pJW009	pRSFDuet-1 P_T7_::*ldhA*::*pdc*::*adhB*::T_T7_; kan^R^	This study
pJW010	pACYCDuet-1 P_T7_::*pct*::*VAAT*::T_T7_; cm^R^	This study
pJW011	pETDuet-1 P_T7_::*pct*::*VAAT*::T_T7_; amp^R^	This study
pJW012	pRSFDuet-1 P_T7_::*pct*::*VAAT*::T_T7_; kan^R^	This study
pJW013	pACYCDuet-1 P_T7_::*ldhA*::*pdc*::*adhB*-P_T7_::*pct*::*VAAT*::T_T7_; cm^R^	This study
pJW014	pETDuet-1 P_T7_::*ldhA*::*pdc*::*adhB*-P_T7_::*pct*::*VAAT*::T_T7_; amp^R^	This study
pJW015	pRSFDuet-1 P_T7_::*ldhA*::*pdc*::*adhB*-P_T7_::*pct*::*VAAT*::T_T7_; kan^R^	This study
pJW016	pACYCDuet-1 P_T7_::*ldhA*::T_T7_-P_T7_::T_T7_; cm^R^	This study
pJW017	pACYCDuet-1 P_T7_::*ldhA*::T_T7_-P_AY1_::T_T7_; cm^R^	This study
pJW018	pACYCDuet-1 P_T7_::*ldhA*::T_T7_-P_AY3_::T_T7_; cm^R^	This study
pJW019	pACYCDuet-1 P_T7_::*ldhA*::T_T7_-P_AY1_::synRBS_pdc#1_::*pdc*::*adhB*::T_T7_; cm^R^	This study
pJW020	pACYCDuet-1 P_T7_::*ldhA*::T_T7_-P_AY1_::synRBS_pdc#2_::*pdc*::*adhB*::T_T7_; cm^R^	This study
pJW021	pACYCDuet-1 P_T7_::*ldhA*::T_T7_-P_AY3_::synRBS_pdc#3_::*pdc*::*adhB*::T_T7_; cm^R^	This study
pJW022	pACYCDuet-1 P_T7_::*ldhA*::T_T7_-P_AY3_::synRBS_pdc#4_::*pdc*::*adhB*::T_T7_; cm^R^	This study
pJW023	pRSFDuet-1 P_T7_::*pct*::T_T7_-P_T7_::T_T7_; kan^R^	This study
pJW024	pRSFDuet-1 P_T7_::synRBS_pct#1_::*pct*::T_T7_-P_T7_::T_T7_; kan^R^	This study
pJW025	pRSFDuet-1 P_T7_::synRBS_pct#2_::*pct*::T_T7_-P_T7_::T_T7_; kan^R^	This study
pJW026	pRSFDuet-1 P_T7_::synRBS_pct#3_::*pct*::T_T7_-P_T7_::T_T7_; kan^R^	This study
pJW027	pRSFDuet-1 P_T7_::synRBS_pct#1_::*pct*::T_T7_-P_T7_::synRBS_VAAT#1_::*VAAT*::T_T7_; kan^R^	This study
pJW028	pRSFDuet-1 P_T7_::synRBS_pct#1_::*pct*::T_T7_-P_T7_::synRBS_VAAT#2_::*VAAT*::T_T7_; kan^R^	This study
pJW029	pRSFDuet-1 P_T7_::synRBS_pct#1_::*pct*::T_T7_-P_T7_::synRBS_VAAT#3_::*VAAT*::T_T7_; kan^R^	This study
pJW030	pRSFDuet-1 P_T7_::synRBS_pct#2_::*pct*::T_T7_-P_T7_::synRBS_VAAT#1_::*VAAT*::T_T7_; kan^R^	This study
pJW031	pRSFDuet-1 P_T7_::synRBS_pct#2_::*pct*::T_T7_-P_T7_::synRBS_VAAT#2_::*VAAT*::T_T7_; kan^R^	This study
pJW032	pRSFDuet-1 P_T7_::synRBS_pct#2_::*pct*::T_T7_-P_T7_::synRBS_VAAT#3_::*VAAT*::T_T7_; kan^R^	This study
pJW033	pRSFDuet-1 P_T7_::synRBS_pct#3_::*pct*::T_T7_-P_T7_::synRBS_VAAT#1_::*VAAT*::T_T7_; kan^R^	This study
pJW034	pRSFDuet-1 P_T7_::synRBS_pct#3_::*pct*::T_T7_-P_T7_::synRBS_VAAT#2_::*VAAT*::T_T7_; kan^R^	This study
pJW035	pRSFDuet-1 P_T7_::synRBS_pct#3_::*pct*::T_T7_-P_T7_::synRBS_VAAT#3_::*VAAT*::T_T7_; kan^R^	This study

**Table 3:** A list of primers used in this study

Primers	Sequences (5′➝3′)
Pyruvate-to-lactyl-CoA module	
DL_0001	CATCATCACCACCATCACTAA
DL_0002	ATGTATATCTCCTTCTTATAGTTAAAC
DL_0032	TAGAAATAATTTTGTTTAACTATAAGAAGGAGATATACATATGAAACTCGCCGTTTATAG
DL_0033	GGGAACCTTTCTCATTATATCTCCTTTTAAACCAGTTCGTTCGGGC
DL_0034	ACGAACTGGTTTAAAAGGAGATATAATGAGAAAGGTTCCCATTAT
DL_0035	GCCGCTCTATTAGTGATGGTGGTGATGATGTCAGGACTTCATTTCCTTCAG
Pyruvate-to-lactate ester module	
DL_0013	GAGCCTCAGACTCCAGCGTA
DL_0014	ATATCAAGCTTGAATTCGTTACCCGG
DL_0015	GGAGGAACTATATCCGGGTAACGAATTCAAGCTTGATATTAATACGACTCACTATAGGG
DL_0016	GTCCAGTTACGCTGGAGTCTGAGGCTC
Upstream module	
JW_0001	GGGCAGCAGCCATCACCATCATCACCACAGCCAGGATCCATGAAACTCGCCGTTTATAGC
JW_0002	CTAAATAGGTACCGACAGTATAACTCATTATATCTCCTTTTAAACCAGTTCGTTCGGGC
JW_0003	CGAAACCTGCCCGAACGAACTGGTTTAAAAGGAGATATAATGAGTTATACTGTCGGTACC
JW_0004	CGCAAGCTTGTCGACCTGCAGGCGCGCCGAGCTCGAATTCTTAGAAAGCGCTCAGGAAG
JW_0005	GGATCCTGGCTGTGGTGATGA
JW_0006	GAATTCGAGCTCGGCGCG
Downstream module	
JW_0007	GTATATTAGTTAAGTATAAGAAGGAGATATACATATGATGAGAAAGGTTCCCATTATTAC
JW_0008	GAAATTATACTGACCTCAATTTTCTCCATTATATCTCCTTTCAGGACTTCATTTCCTTC
JW_0009	AATGGGTCTGAAGGAAATGAAGTCCTGAAAGGAGATATAATGGAGAAAATTGAGGTCAG
JW_0010	CAAATTTCGCAGCAGCGGTTTCTTTACCAGACTCGAGTCAATATCTTGAAATTAGCGTCT
JW_0011	CATATGTATATCTCCTTCTTATACTTAACT
JW_0012	CTCGAGTCTGGTAAAGAAAC
Synthetic operons for upstream module	
JW_0013	GGGAATTGTGAGCGGATAACAATTCCCCAAGGAGATATAATGAAACTCGCCGTTTATAGC
JW_0014	TTATGCTAGTTATTGCTCAGCGGTGGCGGCCGCTCTATTATTAAACCAGTTCGTTCGG
JW_0015	TCTGGAAAAAGGCGAAACCTGCCCGAACGAACTGGTTTAATAATAGAGCGGCCGC
JW_0016	GATTATGCGGCCGTGTACAATACGATTACTTTCTGTTCGATTTCTACCGAAGAAAGGC
JW_0017	CATTATATCTCCTTGGGGAATTGTTATCCGC
JW_0018	TCGAACAGAAAGTAATCGTATTG
JW_0019	AAATTTGACGGCTAGCTCAGTCCTAGGTACAGTGCTAGCATGAGTTATACTGTCGGTACC
JW_0020	GCGTTCAAATTTCGCAGCAGCGGTTTCTTTACCAGACTCGAGTTAGAAAGCGCTCAGGAA
JW_0021	AAATCTGACAGCTAGCTCAGTCCTAGGTATAATGCTAGCATGAGTTATACTGTCGGTACC
JW_0022	CATGCTAGCACTGTACCTAGGACTGAGCTAGCCGTCAAATTTCGATTATGCGGCC
JW_0023	CATGCTAGCATTATACCTAGGACTGAGCTAGCTGTCAGATTTCGATTATGCGGCC
JW_0024	TACAGTGCTAGCAGCTTAGCGACAACCCTAGGCGCTCGCATGAGTTATACTGTCGGTACC
JW_0025	GTATAATGCTAGCTTAGCAGTACCAGGACGTACCGGAGTATGAGTTATACTGTCGGTACC
JW_0026	TAGGTACAGTGCTAGCACTAGGCCTAGCGATTCCGCTAAATGAGTTATACTGTCGGTACC
JW_0027	TATAATGCTAGCAGTTTACCTAGGGCAATAGCGTACCGAATGAGTTATACTGTCGGTACC
JW_0028	CATGCGAGCGCCTAGGGTTGTCGCTAAGCTGCTAGCACTGTACCTAGG
JW_0029	CATTTAGCGGAATCGCTAGGCCTAGTGCTAGCACTGTACCTAGG
JW_0030	CATACTCCGGTACGTCCTGGTACTGCTAAGCTAGCATTATACCTAGG
JW_0031	CATTCGGTACGCTATTGCCCTAGGTAAACTGCTAGCATTATACCTAGG
Synthetic operons for downstream module	
JW_0032	TTATGCTAGTTATTGCTCAGCGGTGGCGGCCGCTCTATTATCAGGACTTCATTTCCTTCA
JW_0033	TGCAGAAGGCTTAATGGGTCTGAAGGAAATGAAGTCCTGATAATAGAGCGGCCGC
JW_0034	GATTATGCGGCCGTGTACAATACGATTACTTTCTGTTCGATTTCTACCGAAGAAAGGC
JW_0035	GATATAGCTCGAACGCGGAAAGAGATGAGAAAGGTTCCCATTATTAC
JW_0036	TCAGGACTTCATTTCCTTCA
JW_0037	GCAACCTATTTTAATCCAAGGAAGATCTAATGAGAAAGGTTCCCATTATTAC
JW_0038	GCAATAACAACTAGGAGAGACGACATGAGAAAGGTTCCCATTATTAC
JW_0039	TAATGGGAACCTTTCTCATCTCTTTCCGCGTTCGAGCTATATCGGGGAATTGTTATCCGC
JW_0040	TGCAGAAGGCTTAATGG
JW_0041	GGAACCTTTCTCATTAGATCTTCCTTGGATTAAAATAGGTTGCGGGGAATTGTTATCCGC
JW_0042	TAATGGGAACCTTTCTCATGTCGTCTCTCCTAGTTGTTATTGCGGGGAATTGTTATCCGC
JW_0043	TAACCAAAACACTAACGCAAGATGGAGAAAATTGAGGTCAGT
JW_0044	AGGGCACGAGGAGGAACCAGTAGAATGGAGAAAATTGAGGTCAGT
JW_0045	GCAACCAACACAACGAGGAGGCATTTAATGGAGAAAATTGAGGTCAGT
JW_0046	TACTGACCTCAATTTTCTCCATCTTGCGTTAGTGTTTTGGTTAGGGGAATTGTTATCCGC
JW_0047	CTCAATTTTCTCCATTCTACTGGTTCCTCCTCGTGCCCTGGGGAATTGTTATCCGC
JW_0048	CTCAATTTTCTCCATTAAATGCCTCCTCGTTGTGTTGGTTGCGGGGAATTGTTATCCGC

#### Construction of pyruvate-to-lactate ester modules

A library of pyruvate-to-lactate ester modules with five divergent AATs was constructed to screen for an efficient AAT for production of lactate esters via two rounds of cloning. First, the pyruvate-to-lactyl-CoA module (pJW001) was constructed by assembling three DNA fragments: (i) the *ldhA* gene, encoding d-lactate dehydrogenase, amplified from *E. coli* MG1655 genomic DNA using the primer pair DL_0032/DL_0033, (ii) the *pct* gene, encoding propionate CoA-transferase, amplified from *Clostridium propionicum* genomic DNA using the primer pair DL_0034/DL_0035, and (iii) the backbone amplified from pETite* using the primer pair DL_0001/DL_0002 [48]. Then, the pyruvate-to-lactate ester modules (pJW002-006) were constructed by assembling three DNA fragments: (i) the pyruvate-to-lactyl-CoA module amplified from pJW001 using the primer pair DL_0032/DL_0014, (ii) the *ATF1* gene amplified from pDL004 for pJW002, the *ATF2* gene amplified from pDL005 for pJW003, the *SAAT* gene amplified from pDL001 for pJW004, the *VAAT* gene amplified from pDL006 for pJW005, or the *atfA* gene amplified from pCT16 for pJW006, using the primer pair DL_0015/DL_0016, and (iii) the backbone amplified from pETite* using the primer pair DL_0013/DL_0002. The genes *ATF1* and *ATF2* are originated from *Saccharomyces cerevisiae* [49], whereas the genes *SAAT*, *VAAT*, and *atfA* are derived from *Fragaria ananassa* [50], *F. vesca* [51], and *Acinetobacter* sp. ADP1 [52], respectively.

#### Construction of a library of upstream and downstream modules with various plasmid copy numbers

A library of upstream and downstream modules was constructed to improve ethyl lactate biosynthesis through a combinatorial pathway optimization strategy using three different plasmids: (i) pACYCDuet-1 (P15A origin of replication), (ii) pETDuet-1 (ColE1 origin), and (iii) pRSFDuet-1 (RSF1030 origin), having the plasmid copy numbers of 10, 40, and 100, respectively [53].

The upstream modules (pJW007-009) were constructed by assembling three DNA fragments: (i) the *ldhA* gene amplified from pJW001 using the primer pair JW_0001/JW_0002, (ii) the ethanol module containing *pdc* and *adhB* genes amplified from pCT24 using the primer pair JW_0003/JW_0004, and (iii) the backbone amplified from pACYCDuet-1 for pJW007, from pETDuet-1 for pJW008, or from pRSFDuet-1 for pJW009 using the primer pair JW_0005/JW_0006.

The downstream modules (pJW010-012) were constructed by assembling three DNA fragments: (i) the *pct* gene amplified from pJW001 using the primer pair JW_0007/JW_0008, (ii) the *VAAT* gene amplified from pJW005 using the primer pair JW_0009/JW_0010, and (iii) the backbone amplified from pACYCDuet-1 for pJW010, pETDuet-1 for pJW011, or pRSFDuet-1 for pJW012 using the primer pair JW_0011/JW_0012.

The combined upstream and downstream modules (pJW013-015) were constructed by assembling two DNA fragments: (i) the upstream module amplified from pJW007 using the primer pair JW_0001/JW_0004 and (ii) the backbone containing the downstream module amplified from pJW010 for pJW013, pJW011 for pJW014, or pJW012 for pJW015 using the primer pair JW_0005/JW_0006.

#### Construction of a library of upstream and downstream modules with various promoters and RBSs

For tighter regulation of biosynthetic pathway of ethyl lactate, we constructed the upstream and downstream modules with tunable promoters and RBSs.

The upstream modules (pJW019-022) were constructed via three rounds of cloning. First, the T7 terminator (T_*T7*_) was added between the multiple cloning site 1 (MCS1) and MCS2 of the pACYCDuet-1 backbone to create the first intermediate plasmid, pJW016, by assembling three DNA fragments: (i) the *ldhA* gene amplified from pJW001 using the primer pair JW_0013/JW_0014, (ii) the linker containing T_*T7*_ terminator amplified from pETite* using the primer pair JW_0015/JW_0016, and (iii) the backbone amplified from pACYCDuet-1 using the primer pair JW_0017/JW_0018. Next, the original T7 promoter (P_*T7*_) in MCS2 of pJW016 was replaced with the P_*AY1*_ (BBa_J23100) promoter and P_*AY3*_ (BBaJ23108) promoter to generate two second-intermediate plasmids, pJW017 and pJW018, respectively, by assembling two DNA fragments: (i) the ethanol module amplified from pCT24 under the P_*AY1*_ promoter for pJW017 or P_*AY3*_ promoter for pJW018 using the primer pair JW_0019/JW_0020 or JW_0021/JW_0020, respectively, and (ii) the backbone amplified from pJW016 using the primer pair JW_0022/JW_0012 or JW_0023/JW_0012, respectively. Last, the final four synthetic operons (pJW019-022) were constructed by assembling two DNA fragments: (i) the ethanol module amplified from pCT24 with the synthetic RBS sequences with predicted translation initiation rates of 0.33 a.u. for pJW019 and pJW021 and 0.03 a.u. for pJW020 and pJW022 using the primer pairs JW_0024/JW_0020, JW_0025/JW_0020, JW_0026/JW_0020, and JW_0027/JW_0020, respectively, and (ii) the backbone amplified from pJW017 for pJW019, pJW017 for pJW020, pJW018 for pJW021, and pJW018 for pJW022 using the primer pairs JW_0028/JW_0012, JW_0029/JW_0012, JW_0030/JW_0012, and JW_0031/JW_0012, respectively. The P_*AY1*_ and P_*AY3*_ promoter sequences were obtained from the iGEM Anderson promoter library (http://parts.igem.org/Promoters/Catalog/Anderson) and the strength of promoters were assigned as P_*AY3*_ = 0.5 × P_*AY1*_. The RBS Calculator v2.0 [54, 55] was used to generate four synthetic RBS sequences with predicted translation initiation rates of 0.33 and 0.03 between the P_*AY1*_ (or P_*AY3*_) promoter and *pdc* start codon (Additional file 2: Figure S3).

The downstream modules (pJW027-035) were constructed via three rounds of cloning. First, the T_*T7*_ terminator was added between MCS1 and MCS2 of the pRSFDuet-1 backbone to generate the first intermediate plasmid, pJW023, by assembling three DNA fragments: (i) the *pct* gene amplified from pJW001 using the primer pair JW_0013/JW_0032, (ii) the linker containing T_*T7*_ terminator from pETite* using the primer pair JW_0033/JW_0034, and (iii) the backbone from pRSFDuet-1 using the primer pair JW_0017/JW_0018. Then, the original RBS in MCS1 of pJW023 was replaced with synthetic RBSs of various strengths to generate the second-intermediate plasmids, pJW024-026, by assembling two DNA fragments: (i) the *pct* gene amplified from pJW001 with the synthetic RBS sequences with predicted translation initiation rates at 90, 9000, or 90000 a.u. for pJW024, pJW025, or pJW026 using the primer pair JW_0035/JW_0036, JW_0037/JW_0036, or JW_0038/JW_0036, respectively, and (ii) the backbone amplified from pJW023 using the primer pair JW_0039/JW_0040 for pJW024, JW_0041/JW_0040 for pJW025, or JW_0042/JW_0040 for pJW026, respectively. Last, the final nine downstream modules (pJW027-035) were constructed by assembling a combination of two DNA fragments: (i) the *VAAT* gene amplified from pDL006 with the synthetic RBS sequences predicted with translation initiation rates of 90, 9000, or 90000 a.u. for pJW027/pJW030/pJW033, pJW028/pJW031/pJW034, or pJW029/pJW032/pJW035 using the primer pair JW_0043/JW_0010, JW_0044/JW_0010, or JW_0045/JW_0010, respectively, and (ii) the backbone amplified from pJW024, pJW025, or pJW026 for pJW027-029, pJW030-032, or pJW033-035 using the primer pair JW_0046/JW_0012, JW_0047/JW_0012, or JW_0048/JW_0012, respectively. The RBS Calculator v2.0 [54, 55] was used to generate six synthetic RBS sequences with predicted translation initiation rates of 90, 9000, and 90000 a.u. between the P_*T7*_ promoter and *pct* (or *VAAT*) start codon (Additional file 2: Figure S3).

### Culture media and conditions

#### Culture media

For molecular cloning, seed cultures, and protein expression analysis, the Luria–Bertani (LB) medium, comprising 10 g/L peptone, 5 g/L yeast extract, and 5 g/L NaCl, was used. For high cell density cultures, pre-cultures of bioreactor batch fermentations, and growth inhibition analysis of lactate esters, the M9 hybrid medium [10] with 20 g/L glucose was used. For bioreactor batch fermentations, the M9 hybrid medium with 50 g/L glucose and 100 µL of antifoam (Antifoam 204, Sigma-Aldrich, MO, USA) was used. 30 µg/mL chloramphenicol (cm), 50 µg/mL kanamycin (kan), and/or 50 µg/mL ampicillin (amp) were added to the media for selection where applicable.

#### High cell density cultures

For seed cultures, 2% (v/v) of stock cells were grown overnight in 5 mL of LB with appropriate antibiotics. For pre-cultures, 1% (v/v) of seed cultures was transferred into 100 mL of LB medium in 500-mL baffled flasks. For main cultures, pre-cultures were aerobically grown overnight (at 37 °C, 200 rpm), centrifuged (4700 rpm, 10 min), and resuspended to yield an optical density measured at 600 nm (OD_600nm_) of 3 in M9 hybrid medium containing appropriate concentration of isopropyl-beta-d-thiogalatopyranoside (IPTG) and antibiotics. The resuspended cultures were distributed into 15-mL polypropylene centrifuge tubes (Thermo Scientific, IL, USA) with a working volume of 5 mL and grown for 24 h (h) on a 75° angled platform in a New Brunswick Excella E25 at 37 °C, 200 rpm. The tubes were capped to generate anaerobic condition. All high cell density culture studies were performed in biological triplicates.

#### pH-controlled bioreactor batch fermentations

pH-controlled bioreactor batch fermentations were conducted with a Biostat B+ (Sartorius Stedim, NY, USA) dual 1.5-L fermentation system at a working volume of 1 L M9 hybrid medium. The seed and pre-cultures were prepared as described in high cell density cultures in LB and M9 hybrid media, respectively. For main cultures, 10% (v/v) of pre-cultures were inoculated into fermentation cultures. During the fermentation, to achieve high cell density, dual-phase fermentation approach [25, 56], aerobic cell growth phase followed by anaerobic production phase, was applied. For the first aerobic phase, the temperature, agitation, and air flow rate were maintained at 37 °C, 1000 rpm, and 1 volume/volume/min (vvm) for 4 h, respectively. Then, the oxygen in the medium was purged by sparing nitrogen gas at 2 vvm for 2 h to generate anaerobic condition. For the subsequent anaerobic phase, 0.5 mM of IPTG was added to induce the protein expression, and the culture temperature and nitrogen flow rate were maintained at 30 °C and 0.2 vvm, respectively. During the fermentation, the pH was maintained at 7.0 with 5 M KOH and 40% H_3_PO_4_. Bioreactor batch fermentation studies were performed in biological duplicates.

#### Growth inhibition analysis of lactate esters

Seed cultures of EcDL002 were prepared as described in high cell density cultures. 4% (v/v) of seed cultures was inoculated into 100 µL of the M9 hybrid media, containing various concentrations (0.5–40 g/L) of lactate esters including ethyl-, propyl-, butyl-, isobutyl-, isoamyl-, or benzyl lactate, in a 96-well microplate. Then, the microplate was sealed with a plastic adhesive sealing film, SealPlate^®^ (EXCEL Scientific, Inc., CA, USA) to prevent evaporation of lactate esters and incubated at 37 °C with continuous shaking using a BioTek Synergy HT microplate reader (BioTek Instruments, Inc., VT, USA). OD_600nm_ was measured at 20-min intervals. Growth inhibition studies of lactate esters were performed twice in biological triplicates (*n *= 6).

### Protein expression and SDS-PAGE analysis

Seed cultures were prepared as described in high cell density cultures. 1% (v/v) of seed cultures subsequently inoculated in 500-mL baffled flasks containing 100 mL of LB medium. Cells were aerobically grown at 37 °C and 200 rpm and induced at an OD_600nm_ of 0.6–0.8 with 0.5 mM of IPTG. After 4 h of induction, cells were collected by centrifugation and resuspended in 100 mM of sodium phosphate buffer (pH7.0) at the final OD_600nm_ of 10. Cell pellets were disrupted using a probe-type sonicator (Model 120, Fisher Scientific, NH, USA) on ice–water mixture. The resulting crude extracts were mixed with 6× sodium dodecyl sulfate (SDS) sample buffer, heated at 100 °C for 5 min, and then analyzed by SDS–polyacrylamide gel electrophoresis (SDS-PAGE, 14% polyacrylamide gel). Protein bands were visualized with Coomassie Brilliant Blue staining.

### Analytical methods

#### Determination of cell concentrations

The optical density was measured at 600 nm using a spectrophotometer (GENESYS 30, Thermo Scientific, IL, USA). The dry cell mass was obtained by multiplication of the optical density of culture broth with a pre-determined conversion factor, 0.48 g/L/OD.

#### High-performance liquid chromatography (HPLC)

Glucose, lactate, acetate, ethanol, isobutanol, isoamyl alcohol, and benzyl alcohol were quantified using the Shimadzu HPLC system (Shimadzu Inc., MD, USA) equipped with the Aminex HPX-87H cation exchange column (BioRad Inc., CA, USA) heated at 50 °C. A mobile phase of 10 mN H_2_SO_4_ was used at a flow rate of 0.6 mL/min. Detection was made with the reflective index detector (RID) and UV detector (UVD) at 220 nm.

#### Gas chromatography coupled with mass spectroscopy (GC/MS)

All esters were quantified by GC/MS. For GC/MS analysis, analytes in the supernatants were extracted with dichloromethane (DCM), containing pentanol as an internal standard, in a 1:1 (v/v) ratio for 1 h at 37 °C, 200 rpm in 15-mL polypropylene centrifuge tubes. After extraction, supernatant–DCM mixtures were centrifuged and 5 μL of DCM extracts were injected into a gas chromatograph (GC) HP 6890 equipped with the mass selective detector (MS) HP 5973. For the GC system, helium was used as the carrier gas at a flow rate of 0.5 mL/min and the analytes were separated on a Phenomenex ZB-5 capillary column (30 m × 0.25 mm × 0.25 μm). The oven temperature was programmed with an initial temperature of 50 °C with a 1 °C/min ramp up to 58 °C. Next, a 25 °C/min ramp was deployed to 235 °C and then finally held a temperature of 300 °C for 2 min to elute any residual non-desired analytes. The injection was performed using a splitless mode with an initial injector temperature of 280 °C. For the MS system, a selected ion monitoring (SIM) mode was deployed to detect analytes.

The SIM parameters for detecting lactate esters were as follows: (i) for pentanol, ions 53.00, 60.00, and 69.00 detected from 5.00 to 7.70 min, (ii) for ethyl lactate, ions 46.00, 47.00, and 75.00 detected from 7.70 to 10.10 min, (iii) for propyl lactate, ions 59.00, 88.00, and 89.00 detected from 10.10 to 11.00 min, (iv) for isobutyl lactate, ions 56.00, 57.00, and 59.00 detected from 11.00 to 11.60 min, (v) for butyl lactate, ions 75.00, 91.00, and 101.00 detected from 11.60 to 12.30 min, (vi) for isoamyl lactate, ions 46.00, 73.00, 75.00 from 12.30 to 14.50 min, and (vii) for benzyl lactate, ions 45.00, 91.00, and 180.00 from 14.50 to 15.08 min. The SIM parameters for detecting acetate esters were as follows: (i) for ethyl acetate, ions 45.00, 61.00, and 70.00 detected from 4.22 to 5.35 min, (ii) for propyl acetate, ions 57.00, 59.00, and 73.00 detected from 5.35 to 6.40 min, (iii) for pentanol, ions 53.00, 60.00, and 69.00 detected from 6.40 to 6.60 min, (iv) for isobutyl acetate, ions 56.00, 61.00, and 73.00 detected from 6.60 to 7.70 min, (v) for butyl acetate, ions 57.00, 71.00, and 87.00 detected from 7.70 to 9.45 min, (vi) for isoamyl acetate, ions 58.00, 70.00, and 88.00 detected from 9.45 to 13.10 min, and (vii) for benzyl acetate, ions 63.00, 107.00, and 150.00 from 13.10 to 15.82 min.

#### Statistics

Statistical analysis was performed with SigmaPlot v.14 using the two-tailed unpaired Student’s *t* test.

## Supplementary information

**Additional file 1: Table S1.** Summary of high cell density cultures of EcJW101, EcJW102, EcJW103, EcJW104, and EcJW105 with addition of glucose and various alcohols after 24 h. The subscripts i and f are referred to the initial (0 h) and final (24 h) time of the culture, respectively. **Table S2.** Summary of titer, specific productivity, and yield of esters in high cell density cultures of EcJW101, EcJW102, EcJW103, EcJW104, and EcJW105 with addition of glucose and various alcohols after 24 h. The acyl acetate and acyl lactate columns correspond to the acyl alcohols added. For example, with the exogenous addition of ethanol, the acyl acetate, and acyl lactate columns represent ethyl acetate, and ethyl lactate, respectively. **Table S3.** (A) Summary of high cell density cultures of EcDL002, EcJW201, and EcJW202 after 24 h. (B) Summary of bioreactor batch fermentation of EcJW201 after 18 h. The subscripts i and f are referred to the initial (0 h) and final (24 h) time of the culture, respectively. **Table S4.** Summary of high cell density cultures of EcJW106-108 and EcJW203-208 with different concentrations of IPTG (0.01, 0.1, or 1.0 mM) after 24 h. The subscripts i and f are referred to the initial (0 h) and final (24 h) time of the culture, respectively. **Table S5.** Summary of titer, specific productivity, and yield of esters in high cell density cultures of EcJW106-108 and EcJW203-208 with different concentrations of IPTG (0.01, 0.1, or 1.0 mM) after 24 h. **Table S6.** Summary of high cell density cultures of EcJW209-212 with or without addition of ethanol (2 or 10 g/L) after 24 h. (A) OD600, pH, glucose, lactate, and ethanol. (B) Titer, specific productivity, and yield of esters. The subscripts i and f are referred to the initial (0 h) and final (24 h) time of the culture, respectively. **Table S7.** Summary of high cell density cultures of EcJW213-221 after 24 h. (A) OD600, pH, glucose, lactate, and ethanol. (B) Titer, specific productivity, and yield of esters. The subscripts i and f are referred to the initial (0 h) and final (24 h) time of the culture, respectively. **Table S8.** Summary of high cell density cultures of EcJW109-117 after 24 h. (A) OD600, pH, glucose, lactate, and ethanol. (B) Titer, specific productivity, and yield of esters. The subscripts i and f are referred to the initial (0 h) and final (24 h) time of the culture, respectively.

**Additional file 2: Figure S1.** Expression of the recombinant enzymes in engineered *E. coli* strains. The positions corresponding to the overexpressed proteins are indicated by arrowheads. Lane M represents protein ladder while lanes T, S, and I are referred to total, soluble, and insoluble proteins, respectively. ①~③, Pyruvate-to-lactate ester module; ④~⑤, Ethanol module; ⑥~⑩, Isobutanol module. Protein sizes were predicted with their amino acids sequences. **Figure S2.** Effect of lactate esters on cell growth. (**A**) Specific growth rates of EcDL002 with or without addition of lactate esters. (B) logP values of characterized lactate esters. The values were obtained from http://www.thegoodscentscompany.com. (**C**–**H**) Growth curves of EcDL002 with or without addition of (**C**) *n*-ethyl lactate (NEL), (**D**) *n*-propyl lactate (NPL), (**E**) *n*-butyl lactate (NBL), (**F**) *i*-butyl lactate (IBL), (**G**) *i*-amyl lactate (IAL), and (**H**) benzyl lactate (BZL). **Figure S3.** Design of (**A**) upstream module and (**B**) downstream module of the ethyl lactate pathway. The RBS Calculator v2.0 software was used to generate synthetic RBS sequences. For the upstream, four synthetic RBS sequences were generated with predicted translation initiation rates at 0.33 and 0.03 between the P*AY1* or P*AY3* promoter and *pdc* start codon. For the downstream, six synthetic RBS sequences were generated with predicted translation initiation rates at 90, 9000, and 90000 a.u. between the P*T7* promoter and *pct* or *VAAT* start codon. **Figure S4.** (**A**) Correlation between ester production and the amount of added ethanol in high cell density cultures of EcJW209-212. (**B**) Correlation between ester production and the RBS strength for VAAT expression in high cell density culture of EcJW213-221.

## Data Availability

Additional files 1 and 2 contain supporting data.

## Abbreviations

LdhA: lactate dehydrogenase
Pct: propionate CoA-transferase
AAT: alcohol acyltransferase
ATF1: alcohol acyltransferase from *Saccharomyces cerevisiae*
ATF2: alcohol acyltransferase from *Saccharomyces cerevisiae*
SAAT: alcohol acyltransferase from *Fragaria ananassa*
VAAT: alcohol acyltransferase from *Fragaria vesca*
AtfA: alcohol acyltransferase from *Acinetobacter* sp. ADP1
OD: optical density
DCW: dry cell weight
SDS-PAGE: sodium dodecyl sulfate–polyacrylamide gel electrophoresis
IPTG: isopropyl β-d-thiogalactopyranoside
MCS: multi-cloning site
RBS: ribosome binding site
au: arbitrary unit
HPLC: high-performance liquid chromatography
GC/MS: gas chromatography coupled with mass spectrometry
SIM: selected ion monitoring
DCM: dichloromethane
rpm: revolutions per minute
v/v: volume per volume
vvm: volume per volume per minute
