# Supplementary material for: Microbial biosynthesis of lactate esters
Source: Biotechnol Biofuels. 2019 Sep 20;12:226. doi: 10.1186/s13068-019-1563-z (PMC6753613; doi:10.1186/s13068-019-1563-z)

Supplementary information

Microbial biosynthesis of lactate esters

Jong-Won Lee[1,2] and Cong T. Trinh[1,2,3,§]

[1]Bredesen Center for Interdisciplinary Research and Graduate Education, University of Tennessee, Knoxville, TN, USA

[2]Center for Bioenergy Innovation, Oak Ridge National Laboratory, Oak Ridge, TN, USA

[3]Department of Chemical and Biomolecular Engineering, University of Tennessee, Knoxville, TN, USA

[§]Corresponding author.

Cong T. Trinh

Dept of Chemical and Biomolecular Engineering

University of Tennessee, Knoxville

1512 Middle Dr., DO#432

Knoxville, TN 37996

ctrinh@utk.edu

Additional Contact Information: Jong-Won Lee: biojwlee@gmail.com

Figure S1. Expression of the recombinant enzymes in engineered *E. coli* strains. The positions corresponding to the overexpressed proteins are indicated by arrowheads. Lane M represents protein ladder while lanes T, S, and I are referred to total, soluble, and insoluble proteins, respectively. ①~③, Pyruvate-to-lactate ester module; ④~⑤, Ethanol module; ⑥~⑩, Isobutanol module. Protein sizes were predicted with their amino acids sequences.

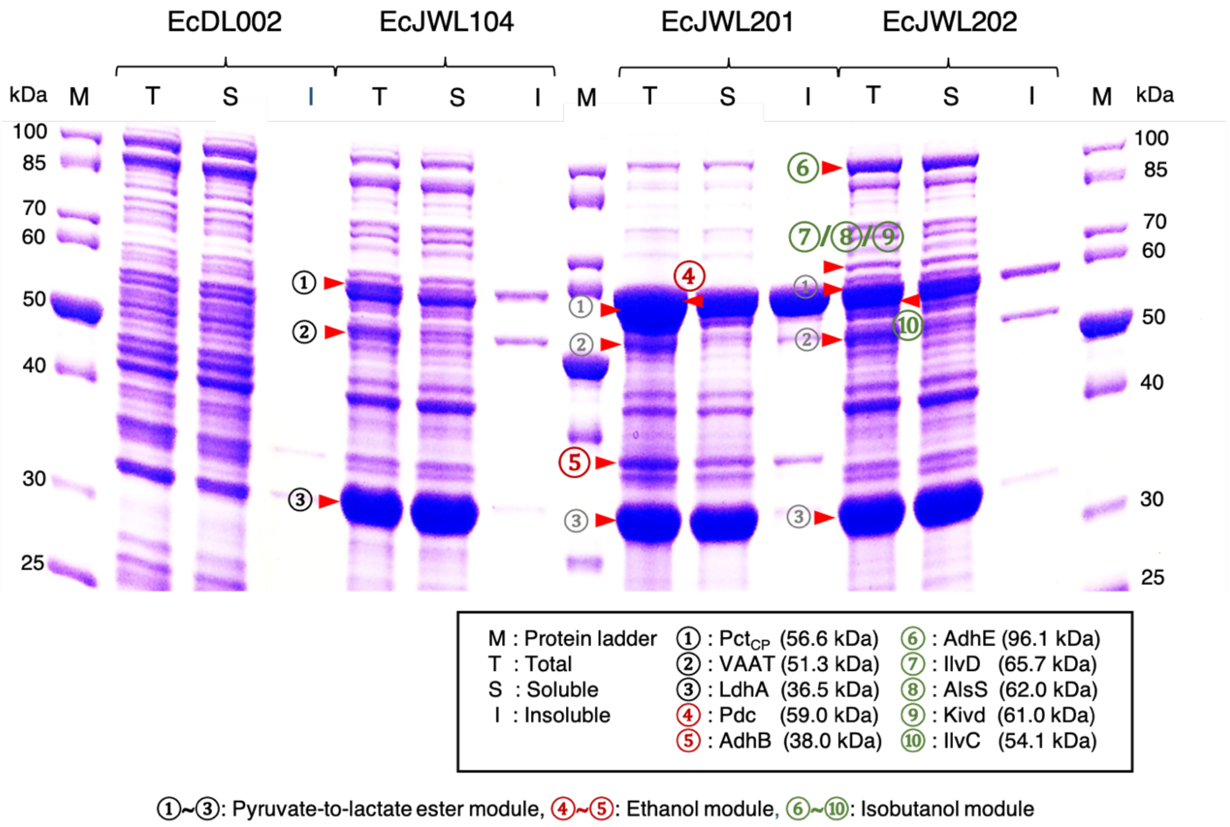

Figure S2. Effect of lactate esters on cell growth. **(A)** Specific growth rates of EcDL002 with or without addition of lactate esters. (B) logP values of characterized lactate esters. The values were obtained from http://www.thegoodscentscompany.com. **(C-H)** Growth curves of EcDL002 with or without addition of **(C)** *n*-ethyl lactate (NEL), **(D)** *n*-propyl lactate (NPL), **(E)** *n*-butyl lactate (NBL), **(F)** *i*-butyl lactate (IBL), **(G)** *i*-amyl lactate (IAL), and **(H)** benzyl lactate (BZL).

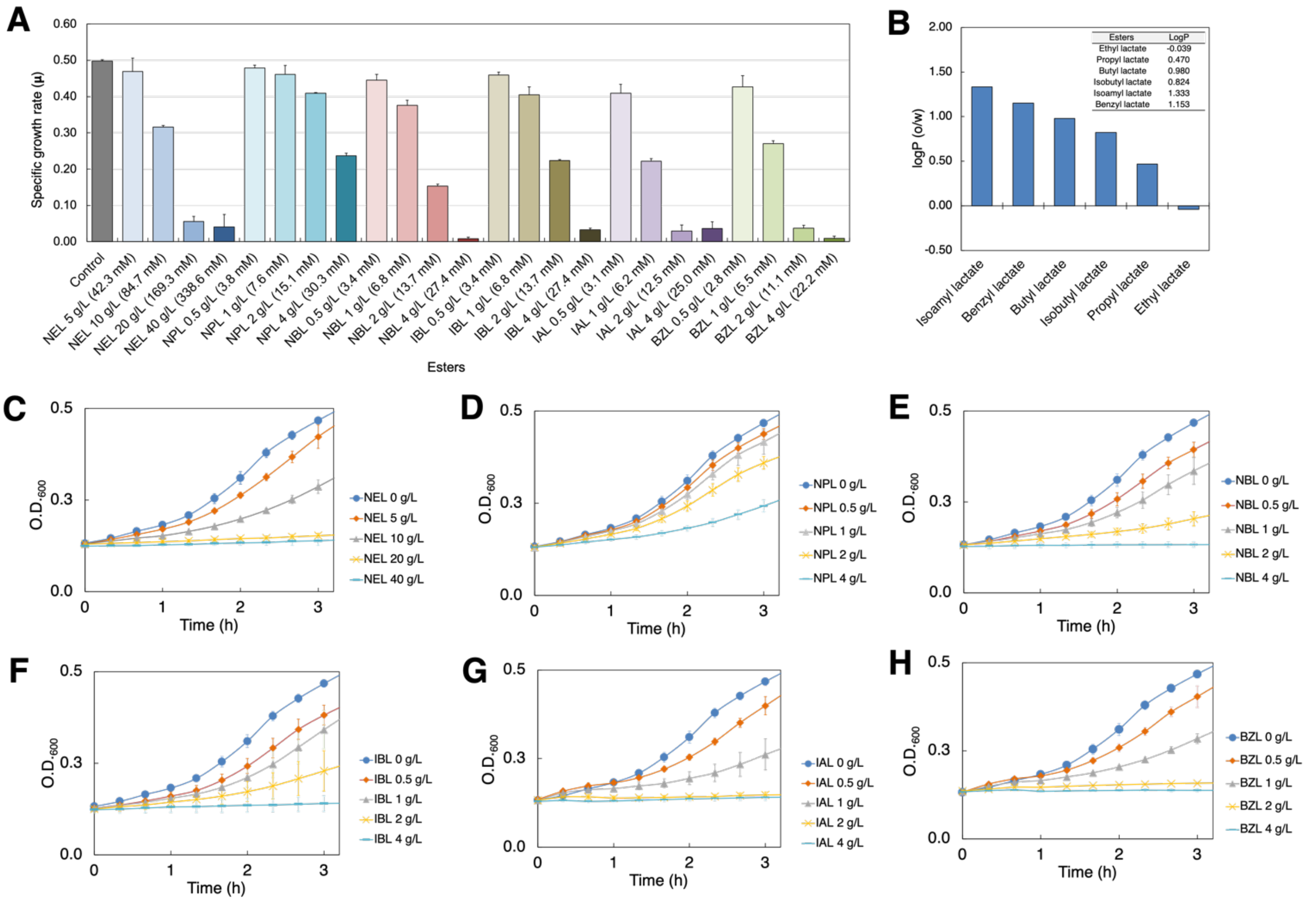

Figure S3. Design of **(A)** upstream module and **(B)** downstream module of the ethyl lactate pathway. The RBS Calculator v2.0 software was used to generate synthetic RBS sequences. For the upstream, four synthetic RBS sequences were generated with predicted translation initiation rates at 0.33 and 0.03 between the P_{AY1} or P_{AY3} promoter and *pdc* start codon. For the downstream, six synthetic RBS sequences were generated with predicted translation initiation rates at 90, 9000, and 90000au between the P_{T7} promoter and *pct* or *VAAT* start codon.

A

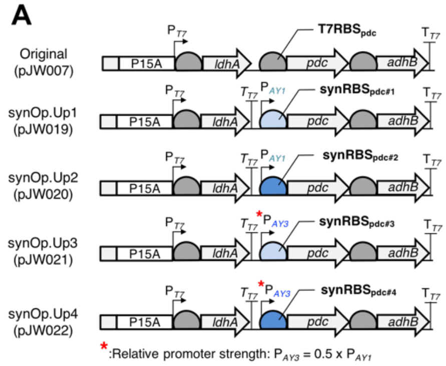

RBS ID	Sequences	Predicted translation initiation rate (au)	Relative translation initiation rate (au)	Length (bp)
**$T7RBS_{pdc}$**	AAGGAGATATA	33.11	1	11
**$synRBS_{pdc\#1}$**	TTAGCAGTACCAGGACGTACCGGAGT	0.33	10^{-2}	27
**$synRBS_{pdc\#2}$**	AGTTTACCTAGGGCAATAGCGTACCGA	0.03	10^{-3}	27
**$synRBS_{pdc\#3}$**	AGCTTAGCGACAACCCTAGGCGCTCGC	0.35	10^{-2}	27
**$synRBS_{pdc\#4}$**	ACTAGGCCTAGCGATTCCGCTAA	0.03	10^{-3}	23

P_{AY1} (BBa_J23100) and P_{AY3} (BBa_J23108) were obtained from iGEM Andersen promoter library

B

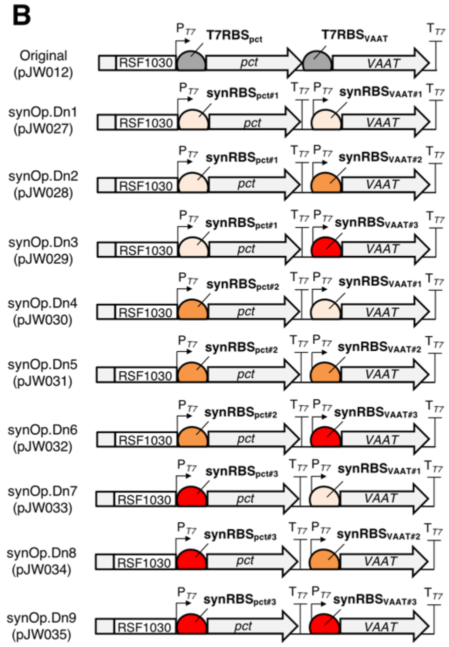

RBS ID	Sequences	Predicted translation initiation rate (au)	Relative translation initiation rate (au)	Length (bp)
**$T7RBS_{pct}$**	AAGGAGATATA	9417.53	10^{2}	11
**$synRBS_{pct\#1}$**	GATATAGCTCGAACGCGGAAAGAG	84.56	10^{0}	24
**$synRBS_{pct\#2}$**	GCAACCTATTTTAATCCAAGGAAGATCT A	8695.18	10^{2}	29
**$synRBS_{pct\#3}$**	GCAATAACAACTAGGAGAGACGAC	95382.72	10^{3}	24
**$T7RBS_{VAAT}$**	AAGGAGATATA	87.01	1	11
**$synRBS_{VAAT\#1}$**	TAACCAAAACACTAACGCAAG	93.02	10^{0}	22
**$synRBS_{VAAT\#2}$**	AGGGCACGAGGAGGAACCAGTAGA	8228.14	10^{2}	24
**$synRBS_{VAAT\#3}$**	GCAACCAACACAACGAGGAGGCATTTA	93714.75	10^{3}	27

Figure S4. (A) Correlation between ester production and the amount of added ethanol in high cell density cultures of EcJW209-212. **(B)** Correlation between ester production and the RBS strength for VAAT expression in high cell density culture of EcJW213-221.

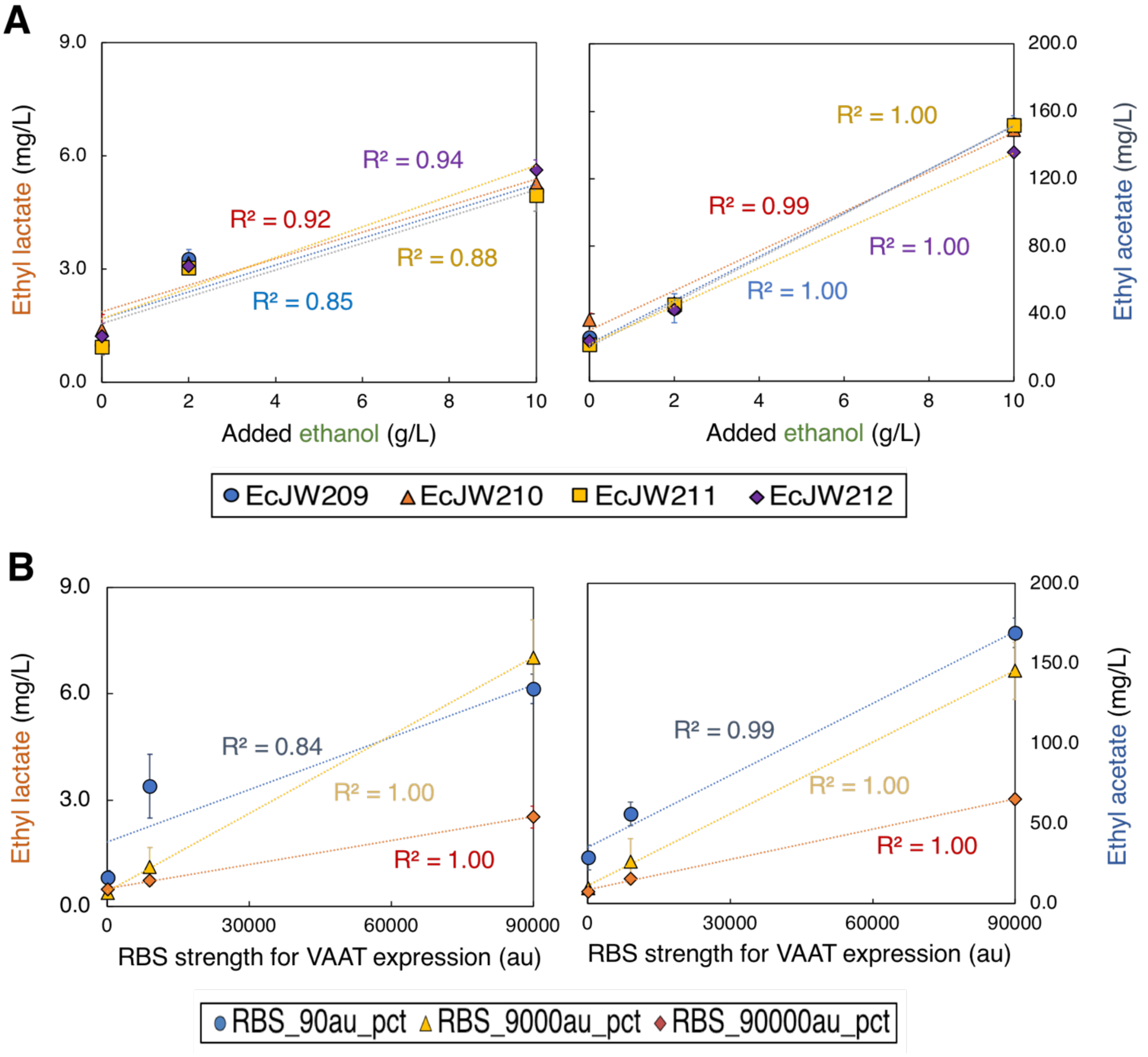

Supplement: Supplementary file 2 — Additional file 2: Figure S1. Expression of the recombinant enzymes in engineered E. coli strains. The positions corresponding to the overexpressed proteins are indicated by arrowheads. Lane M represents protein ladder while lanes T, S, and I are referred to total, soluble, and insoluble proteins, respectively. ①~③, Pyruvate-to-lactate ester module; ④~⑤, Ethanol module; ⑥~⑩, Isobutanol module. Protein sizes were predicted with their amino acids sequences. Figure S2. Effect of lactate esters on cell growth. (A) Specific growth rates of EcDL002 with or without addition of lactate esters. (B) logP values of characterized lactate esters. The values were obtained from http://www.thegoodscentscompany.com. (C–H) Growth curves of EcDL002 with or without addition of (C) n-ethyl lactate (NEL), (D) n-propyl lactate (NPL), (E) n-butyl lactate (NBL), (F) i-butyl lactate (IBL), (G) i-amyl lactate (IAL), and (H) benzyl lactate (BZL). Figure S3. Design of (A) upstream module and (B) downstream module of the ethyl lactate pathway. The RBS Calculator v2.0 software was used to generate synthetic RBS sequences. For the upstream, four synthetic RBS sequences were generated with predicted translation initiation rates at 0.33 and 0.03 between the PAY1 or PAY3 promoter and pdc start codon. For the downstream, six synthetic RBS sequences were generated with predicted translation initiation rates at 90, 9000, and 90000 a.u. between the PT7 promoter and pct or VAAT start codon. Figure S4. (A) Correlation between ester production and the amount of added ethanol in high cell density cultures of EcJW209-212. (B) Correlation between ester production and the RBS strength for VAAT expression in high cell density culture of EcJW213-221. [file 13068_2019_1563_MOESM2_ESM.pdf]
